# Smiling and first impressions in ad hoc entrustment decisions: An avatar-based simulation study

**DOI:** 10.3205/zma001810

**Published:** 2026-02-17

**Authors:** Moritz Bauermann, Ann-Kathrin Schindler, Marco Kuchenbaur, Jasmin Rühl, Patrick Reinert, Miriam Kunz, Sarah Friedrich-Welz, Elisabeth André, Thomas Rotthoff

**Affiliations:** 1 University of Augsburg, Faculty of Medicine, Medical Didactics and Education Research Augsburg (DEMEDA), Augsburg, Germany; 2 University of Augsburg, Faculty of Mathematics, Natural Sciences, and Materials Engineering, Mathematical Statistics and Medicine, Augsburg, Germany; 3 University of Augsburg, Faculty of Medicine, Institute of Theoretical Medicine, Medical Psychology and Sociology, Augsburg, Germany; 4 University of Augsburg, Faculty of Applied Computer Science, Human-Centered Artificial Intelligence, Augsburg, Germany

**Keywords:** entrustable professional activities, trust, entrustment, non-verbal expressions, first impression, medical education, Duchenne smile, avatars

## Abstract

**Objective:**

We aimed to examine how neutral and smiling facial expressions, as indicators of non-verbal first impressions, influence ad hoc entrustment and perceptions of trustworthiness in a person in medical scenarios that demand entrustment decisions.

**Methodology:**

Within the framework of entrustable professional activities (EPA), we tested three hypotheses in an online study using avatar simulations. As a pilot study and for reasons of sample accessibility, 268 medical students (67.2% female) read 36 narrative clinical entrustment situations in a randomized order. The participants then had to decide whether to entrust the clinical task to fellow students who appeared in short videos as standardized avatars. For each scenario, two avatars were presented and randomly matched for different facial expressions (Duchenne smile (DS), neutral), genders, and morphologies. The participants also assessed the avatars’ perceived trustworthiness.

**Results:**

Entrustment and trustworthiness were positively correlated (H1). However, when utilizing mixed-effects models, no significant differences were found between the avatars that displayed a DS and those with a neutral expression regarding positive entrustment decisions (H2) or trustworthiness ratings (H3).

**Conclusion:**

We found that trustworthiness was linked to entrustment, which supports previous findings on trustworthiness as a person-related prerequisite for entrustment decisions. Internalized patterns of the DS did not influence the medical students’ entrustment decisions in the first-impression situations. This suggests that in ad hoc medical entrustment scenarios, factors such as context or other aspects of a trustee’s first impression may shape trustors’ decisions.

## Zusammenfassung

**Zielsetzung:**

Untersucht wurde der Einfluss neutraler und lächelnder Gesichtsausdrücke als Indikatoren des nonverbalen ersten Eindrucks. Es wurde analysiert, wie diese Indikatoren das Ad-hoc-Anvertrauen sowie die Wahrnehmung von Vertrauenswürdigkeit in medizinischen Entscheidungssituationen beeinflussen.

**Methodik:**

Im Rahmen des Konzepts der Entrustable Professional Activities (EPA) wurde eine Online-Studie mit Avataren durchgeführt und drei Hypothesen überprüft. Als Pilotstudie und aufgrund der Stichprobenzugänglichkeit nahmen 268 Medizinstudierende (67,2% weiblich) teil. Sie lasen 36 narrative klinische Anvertrauenssituationen, die ihnen in randomisierter Reihenfolge präsentiert wurden. Anschließend sollten die Teilnehmenden entscheiden, ob sie die jeweilige klinische Aufgabe Mitstudierenden anvertrauen würden, die in kurzen Videos durch Avatare dargestellt wurden. Für jedes Szenario wurden zwei Avatare gezeigt, die zufällig in Gesichtsausdruck (Duchenne-Lächeln (DL), neutral), Geschlecht und Morphologie variierten. Neben der Anvertrauensentscheidung bewerteten die Teilnehmenden, wie vertrauenswürdig die Avatare auf sie wirkten.

**Ergebnisse:**

Anvertrauen und Vertrauenswürdigkeit korrelierten positiv (H1). Die Analyse mittels Mixed-Effect-Modellen zeigte jedoch keine signifikanten Unterschiede zwischen Avataren mit DL und solchen mit neutralem Gesichtsausdruck für positiv getroffene Anvertrauensentscheidungen (H2) oder die wahrgenommene Vertrauenswürdigkeit (H3).

**Schlussfolgerung:**

Der Zusammenhang zwischen Vertrauenswürdigkeit mit Anvertrauen bestätigt vorhandene Erkenntnisse, die Vertrauenswürdigkeit als wichtige personenbezogene Voraussetzung für Anvertrauensentscheidungen beschreiben. Die internalisierten Muster eines DL beeinflussten die Anvertrauensentscheidungen der Medizinstudierenden innerhalb der Ersteindruckssituationen jedoch nicht. Ersteres unterstützt die Annahme, dass in medizinischen Ad-hoc-Anvertrauenssituationen, Faktoren wie der Kontext oder andere Aspekte des ersten Eindrucks eines Trustees die Entscheidung des Trustors beeinflussen könnten.

## 1. Introduction

In the medical field, trust is crucial, as the core of everyday healthcare practices revolves around human encounters, which necessitate daily trust [1]. Trust typically involves one person (the trustor) placing their trust in another (the trustee). As a process, trust can be further differentiated into *trustworthiness*, which is a mental state encompassing a trustor’s readiness to be vulnerable with a trustee, and *entrustment*, which is the act of trusting someone that usually happens once trustworthiness has been established [2]. Examples of entrustment in the medical context are intra- and interprofessional entrustment between colleagues, between patients and physicians, and between supervisors and their trainees [3], [4], [5], [6], [7]. Entrustment fosters an environment that is conducive for professional growth and effective patient care, both of which significantly bolster treatment efficacy and patient well-being [3], [4], [5], [6], [7]. The principle of trust is deeply integrated into the fabric of medical education, particularly through the entrustable professional activities (EPA) framework. This framework entails the allocation of clinical responsibilities from educator to learner and exemplifies the integral role of trust in the development of medical competence [8]. Such entrustment decisions are often made on an ad hoc situational basis, without much information regarding the trustee, as highlighted by ten Cate [9]. In this study, we postulated that for such ad hoc entrustment decisions, the first impressions of a trustee can have a significant impact.

It is well established in contexts other than medical education that a person quickly decides whether to trust a stranger based on the stranger’s first facial expression [10], [11]. Based on the theory of trait inference mapping, a person activates their face space (i.e., storage of facial representations) and trait space (i.e., storage of behavioral representations) when first seeing another person [12]. Both coordinates are matched, and with repetition, patterns are learned [12]. A Duchenne smile (DS) has been shown as one relevant facial expression that can influence this attribution process [11]. Named after Duchenne de Boulogne, who initially described it, this smile involves the zygomaticus major and orbicularis oculi muscles [13]. A DS is associated with happiness on the part of its sender and is perceived more positively than a neutral expression [14]. While debate continues about its ambiguous effects [15], [16], a DS is considered a sign of authenticity regardless of the underlying emotion [17], [18]. 

Based on this rationale, we investigated three hypotheses. First, we considered trustworthiness and entrustment to be positively correlated (H1). Second, we hypothesized that trustees displaying a DS as a non-verbal first impression receive more positive entrustment decisions (H2) and heightened trustworthiness ratings (H3) compared to those with neutral facial expressions. The hypotheses were tested in a cost-effective and time-efficient manner through the use of simulated avatars in an online survey and the selection of medical students as participants. 

## 2. Methods

### 2.1. Design 

**Figure 1:**
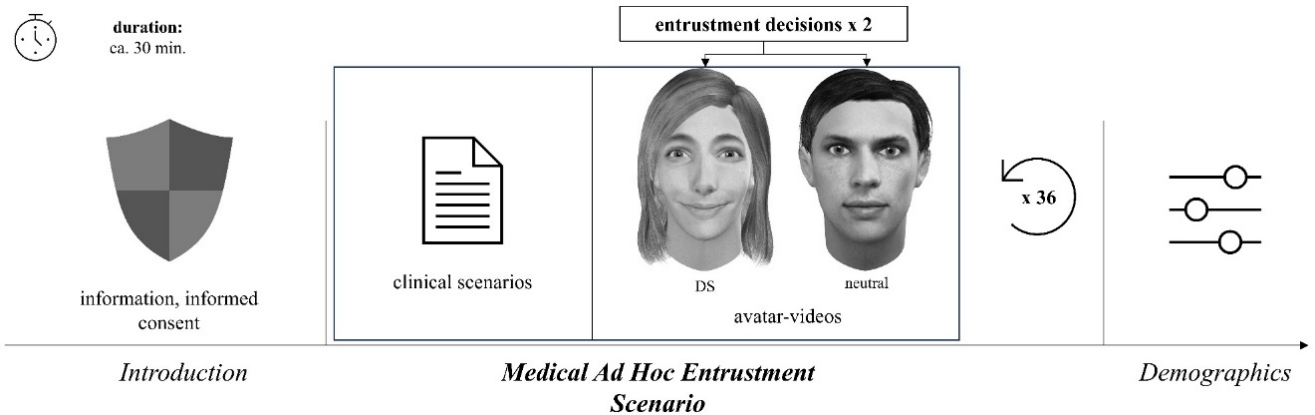
Study design [20] DS=Duchenne smile

The online survey was conducted using the online survey tool soSci [19], in May and June 2025. The study was approved by the ethics committee of LMU Munich (Project: 22-0367_1). A within-subject design was applied in the study, which was carried out as illustrated in figure 1 (Fig. 1).

#### 2.1.1. Introduction

First, the participants were provided with comprehensive information about the study and the protection of their data. They then gave their informed consent and accepted a data protection waiver. Next, they read the standardized instructions for the study. 

#### 2.1.2. Medical ad hoc entrustment scenarios

The participants were presented with 36 consecutive medical ad hoc entrustment scenarios in a randomized order. They received two short breaks – after the 12^th^ and 24^th^ scenarios – to keep them focused [20]. All the clinical scenarios were written by an experienced physician. He formulated six of the scenarios based on typical medical situations that are generally considered suitable for undergraduate medical students in clinical environments. For example, the entrustment situation of blood sampling concerned drawing a daily blood sample in an internal medicine ward. In this scenario, the ward physician offers the student (participant) the opportunity to attend a gastroscopy instead of taking the sample. The participants then had to decide whether to entrust the task to a fellow student (the trustee). The six scenarios served as the basis for slight adaptations and variations to produce a total of 18 scenarios, which were each presented twice. All the scenarios were presented randomly and intended for the enforcement of an entrustment decision. 

The trustees were represented by avatars. After each scenario, the participants viewed two 5-second videos of these avatars. To minimize potential distractions and help the participants focus on the avatars’ facial expressions, only the avatars’ heads were designed [21]. The avatars were randomized in terms of gender, morphological features, and hairstyles. To test H2 and H3, the facial expressions were standardized across the avatars, which displayed either a DS or a neutral expression. The facial expressions were animated using FACSGen 2022 [22]. FACSGen is software based on the Facial Action Coding System, which describes facial muscular movements as action units (AUs) [23]. The main benefits of manipulation via AUs are clarity and reproducibility [24]. We created the two expressions using the AUs shown in table 1 (Tab. 1).

To meet the estimated statistical power (see Section 2.2), a total of 18 female and 18 male avatars were created. Each avatar was presented once with a DS and once with a neutral expression. Consequently, each participant provided 72 entrustment decisions (H2) and trustworthiness ratings (H3). After presenting the trustee as an avatar, the participants were asked whether they would entrust the medical task described in the scenario to the trustee (response options dichotomously: yes/no). In addition, they rated the avatar trustee’s trustworthiness on a 4-point Likert scale ranging from 0 (not at all) to 3 (very). 

#### 2.1.3. Demographics

The participants provided their demographic data (gender, age, and semester) for the sample description.

### 2.2. Sample 

We recruited 268 volunteer medical students (67.2% female; age: n_20–25y_=249; n_>26y_=19; semester: n_2nd semester_=149, n_4th semester_=116, n_>4_=3) from the second and fourth semesters, since the clinical scenarios were designed to be suitable for students from different semesters. At the beginning of the survey period, potential participants received an email invitation outlining the study’s aims, data protection measures, and details regarding compensation. A QR code that linked directly to the online survey was also included. The participants could take part in the study at any time and in any place using a digital device of their choice. The sample was therefore self-selected, and no further randomization took place. 

To account for potential random effects of the participants and avatars in the subsequent mixed-model analyses, we conducted a simulation-based power analysis using the *lme4* and *simr* packages in R [25], [26]. The model included 72 avatars per participant as repeated measures. This number was chosen for its practicality, as it allowed for an even split across the 36 entrustment narratives, the associated facial expressions, and the genders of the avatars. We had previously collected data from 26 participants. Based on this sample, the estimated statistical power was approximately 23%. To determine the sample size needed to reliably detect the observed effect at a significance level of .05, we ran 1000 simulation analyses. These simulations indicated that, under the same assumptions, a sample size of about 200 participants would be required to achieve 80% power. Random intercepts for both the participants and avatars were included in the model. This power analysis was based on the entrustment decisions, so the estimated sample size should be viewed as exploratory with regard to trustworthiness. 

### 2.3. Analyses

For H1 (correlations between trustworthiness and entrustment), a partial Spearman correlation was conducted using the *ppcor* [27], package in R while controlling for the participants and avatars. The classification of effect strengths was based on the work of Cohen, with small=.10, medium=.30, and large=.50 [28].

We tested H2 (dichotomous entrustment decision) using a logistic mixed-effects model with a binomial link. The initial H2-0 model included an overall intercept (β_0_) and random intercepts for the participants (*u_0i_*) as well as the avatars (*v_0j_*), where *u_0i_* and *v_0j_* included the genders of both the participants and avatars, respectively, to account for their inherent randomness, with no predictions added:

logit (Ρ(entrustment_ij_=1))=β_0_+*u_0i_*+*v_0j_*

In the next model (H2-1), we added the facial expression as a fixed-effect predictor (β_1_ · facial_expression_ij_) to test whether the presence of a DS would influence the probability of a positive entrustment decision beyond the baseline participant and avatar randomness: 

logit (Ρ(entrustment_ij_=1))=β_0_+β_1_ · facial_expression_ij_+*u_0i_*+*v_0j_*

For H3 (dimensional perceived trustworthiness), we estimated a cumulative link mixed-effects model with trustworthiness ratings as the dependent variable utilizing the ordinal [29] package in R. For the H3 model, an overall intercept (β_0_) and, again, random intercepts for the participants (*u_0i_*) and avatars (*v_0j_*) were included. κ indexed the thresholds between the ordinal trustworthy categories. As before, facial expression (β_0_ · facial_expression_ij_) was included as a fixed effect to test whether the presence of a DS would influence trustworthiness ratings:

logit (Ρ(trustworthiness_ij_≤Κ))=θκ–(β_0_ · facial_expression_ij_+*u_0i_*+*v_0j_*)

## 3. Results

The medical students’ overall positive entrustment decisions for the avatar trustees varied from 0 to 72 (max. possible: 72), with a median of 40 (IQR=20.0). The overall perceived trustworthiness varied between 0 and 3, with a median of 1.66 (IQR=.56). 

### 3.1. Relationship between entrustment and trustworthiness 

**Figure 2:**
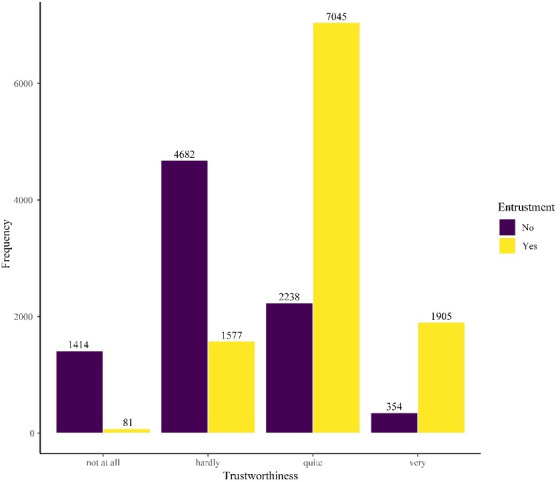
Relationship between trustworthiness and entrustment (n=19,296)

Regarding H1, trustworthiness was positively asociated with entrustment (*ρ*=.55, *p*<.001). As shown in figure 2 (Fig. 2), the majority of the trustees simulated by avatars were perceived as quite trustworthy, which coincided with more positive entrustment decisions (shown in yellow). The same pattern held for the “very” trustworthy label. In contrast, the negative end of the trustworthiness scale showed the reverse pattern, with lower trustworthiness ratings associated with lower entrustment. 

### 3.2. Entrustment 

**Figure 3:**
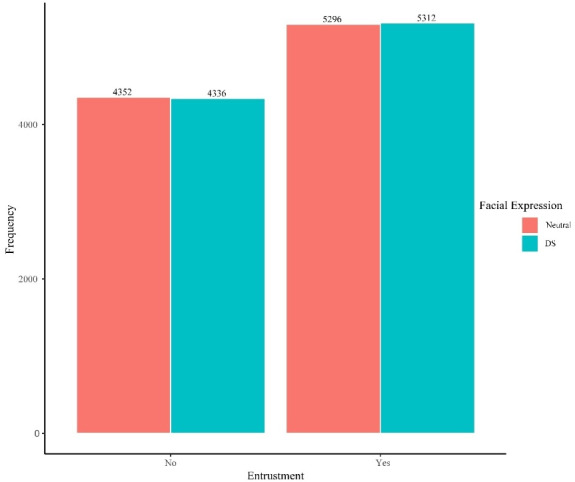
Distribution of entrustment decisions by facial expression (n=19,296) DS=Duchenne smile

In terms of H2, for the H2-0 model (without predictors), the intercept was 1.29, and the intercept variance was at .002, which indicated a higher probability of a positive entrustment decision. The results for the H2-1 model are presented in table 2 (Tab. 2) and illustrated in figure 3 (Fig. 3). When testing facial expression as a predictor, H2 was not supported – a DS did not increase the likelihood of positive entrustment, as indicated by an (OR) of 1.01 (95% CI [0.95–1.07], *p*=.796). The participants had randomly varying entrustment behaviors (τ_00participant_=1.68), whereas the avatars did not cause any random effects (τ_00avatar_=0.00). 

### 3.3. Trustworthiness

**Figure 4:**
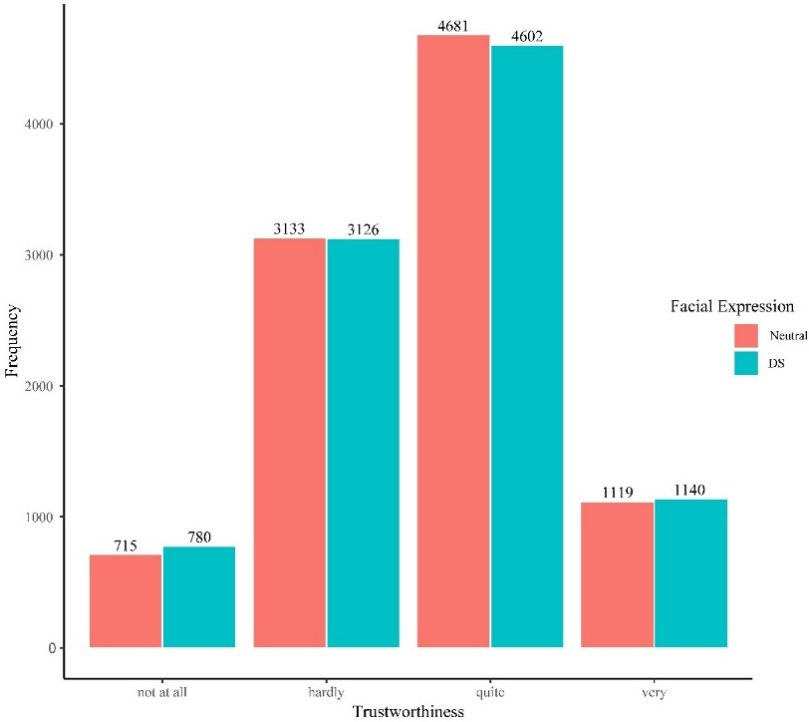
Distribution of trustworthiness by facial expression (n=19,296) DS=Duchenne smile

When testing facial expression as a predictor, H3 was not supported – a DS did not increase the assessed trustworthiness positively, as indicated by an OR of 0.97 (95% CI [0.92–1.02], *p*=.274). Once again, the participants had randomly varying assessments of trustworthiness (τ_00participant_=0.90), whereas the avatars did not cause any random effects (τ_00avatar_=0.00). The conditional Pseudo-R^2^ (0.215) showed that only a small portion of the overall variance in the assessed trustworthiness could be explained by the fixed and random effects that were included in this model. The results for H3 are presented in table 3 (Tab. 3) and illustrated in figure 4 (Fig. 4).

## 4. Discussion

In this study, we examined how different types of facial expressions (DS, neutral) – as one indicator for non-verbal first impressions – influence entrustment and the perception of trustworthiness in another person in the context of ad hoc medical entrustment decisions. First, we found that higher trustworthiness of a simulated trustee was positively correlated with ad hoc entrustment. This finding extends the widely accepted understanding that people can only entrust someone if they consider the person trustworthy [2], [30]. This assessment of entrustment and trustworthiness is a crucial factor in thorough EPA evaluations [31], [32]. Furthermore, trustworthiness may be especially important when supervisors need to make ad hoc entrustment decisions in medical settings [33].

Second, we did not find support for H2 and H3, as a DS had no impact on entrustment and trustworthiness. Although the positive influence of a DS is indicated in other contexts [14], [34], we were unable to replicate these effects in this study. This may be due to the highly context-sensitive nature of trust in medical settings. As Holzhausen noted, entrustment in clinical contexts often means assigning tasks and responsibilities, which is an inherently risky process [35]. Contextual variability in medical settings is recognized as a potential determinant of trustors’ entrustment decisions [36], [37]. The trustor must therefore take context-specific factors, such as patient safety, time pressure, and personnel resources, into account to make informed decisions [35]. In conclusion, the context of a clinical scenario could influence ad hoc entrustment and perceived trustworthiness. 

The limitations of this study included the self-selection inherent in the sample and the choice of avatars. Given the current rapid development of human-like avatars, more realistic-looking avatars, such as Epic Game’s MetaHumans [38], are available, but restrictions on compatibility are present in the FACS system. This limited the potential of reproducibility and the standardization of the facial expressions [24] we were able to apply with our choice of avatars. Additionally, far more observable indicators of real first impressions (confidence in appearance, tone of voice) [39], [40], are available beyond DS or a neutral facial expression. Lastly, we did not investigate the extent to which sympathy for the different avatars played a role. The absence of random effects due to the avatars, however, suggests that sympathy was unlikely to play a major role.

## 5. Conclusion

In this online study with its highly simulated setting, we found trustworthiness was linked to entrustment. Our findings therefore support those of previous studies on trustworthiness as the person-related prerequisite for an entrustment decision. When the avatars were tested in our sample of medical students, the students’ internalized patterns regarding the DS did not influence their entrustment decisions in the first impression situations. This suggests that in ad hoc medical entrustment scenarios, factors such as context or other aspects of a trustee’s first impression may shape trustors’ decisions. Future research could focus on exploring the influence of these other factors of non-verbal first impressions on ad hoc entrustment decisions in relation to EPAs in medical education.

## Notes

### Author contributions

- *Moritz Bauermann* made significant contributions to the conceptualization of the study design and methodology, acquisition of the participants, investigation, implementation of the data analyses and evaluation, visualization, writing (first draft and editing), and incorporation of feedback.
- *Ann-Kathrin Schindler* made significant contributions to the conceptualization of the study design and methodology and contributed considerably to the data analyses and evaluation, writing (review and feedback), and funding acquisition.
- *Marco Kuchenbauer* made significant contributions to the data analyses and evaluation and writing (review and feedback).
- *Jasmin Rühl* made significant contributions to the conceptualization of the study design and methodology and contributed considerably to the data analyses and evaluation and writing (review and feedback).
- *Patrick Reinert* made significant contributions to the conceptualization of the study design and methodology and contributed considerably to the data analyses and evaluation and writing (review and feedback).
- *Miriam Kunz *made significant contributions to the conceptualization of the study design and methodology, the data analyses and evaluation, writing (review and feedback), and funding acquisition.
- *Sarah Friedrich-Welz* made significant contributions to the conceptualization of the study design and methodology, data analyses and evaluation, and writing (review and feedback).
- *Elisabeth André* made significant contributions to the writing and reviewing of the manuscript as well as the acquisition of funding.
- *Thomas Rotthoff *made significant contributions to the conceptualization of the study design and methodology, data analyses and evaluation, creation of the clinical scenarios, writing (review and feedback), supervision, and funding acquisition.

### Authors’ ORCIDs

- Moritz Bauermann: [0009-0000-5786-0149]
- Ann-Kathrin Schindler: [0000-0002-2293-2357]
- Marco Kuchenbaur: [0000-0003-0280-1541]
- Jasmin Rühl: [0000-0002-1721-0640]
- Miriam Kunz: [0000-0002-0740-6738]
- Sarah Friedrich: [0000-0003-0291-4378]
- Elisabeth André: [0000-0002-2367-162X]
- Thomas Rotthoff: [0000-0002-5171-5941]

### Ethical approval and consent to participate

The Ethics Committee of the Medical Faculty, LMU Munich, approved this study as ethically sound (Project: 22-0367_1). The methods applied in this study adhered to the principles of the Declaration of Helsinki.

Prior to conducting the study, all the participants were informed about the study’s intent and data security. They subsequently gave their informed consent and accepted a data protection waiver. 

### Consent for publication

The data protection waiver also included consent for publication.

### Availability of the data and materials

The datasets used and/or analyzed during the study are available from the corresponding author on reasonable request. 

## Funding

This work was funded by the project “Facilitating Competence Development through Digital Authentic, and Feedback-Based Learning Scenarios – KodiLL” by Stiftung Innovation in der Hochschullehre (FBM2020-262/2021).

## Acknowledgements

We would like to sincerely thank all the participating students for their contributions.

## Competing interests

The authors declare that they have no competing interests. 

## Figures and Tables

**Table 1:**
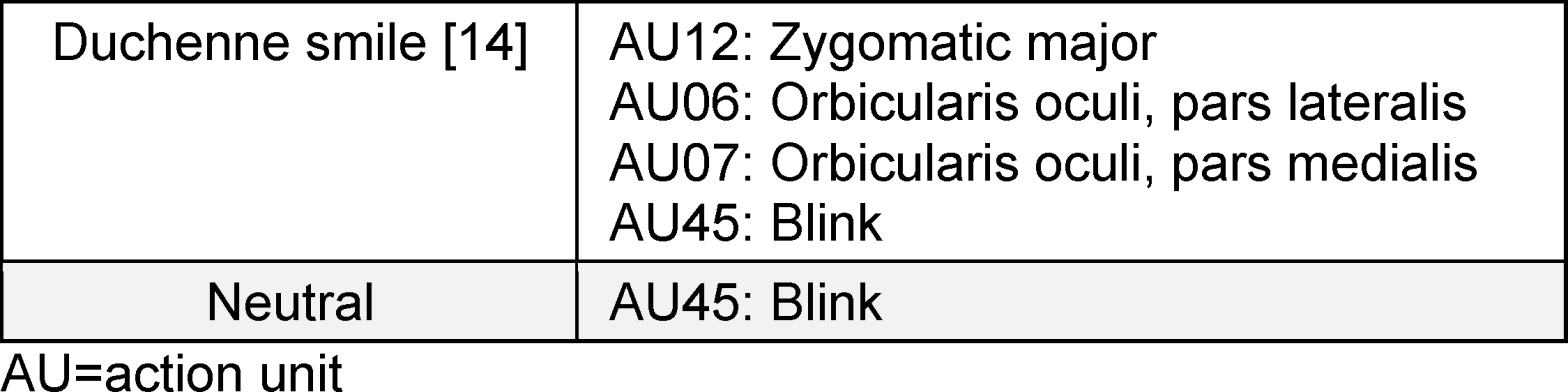
Action units of the expressions

**Table 2:**
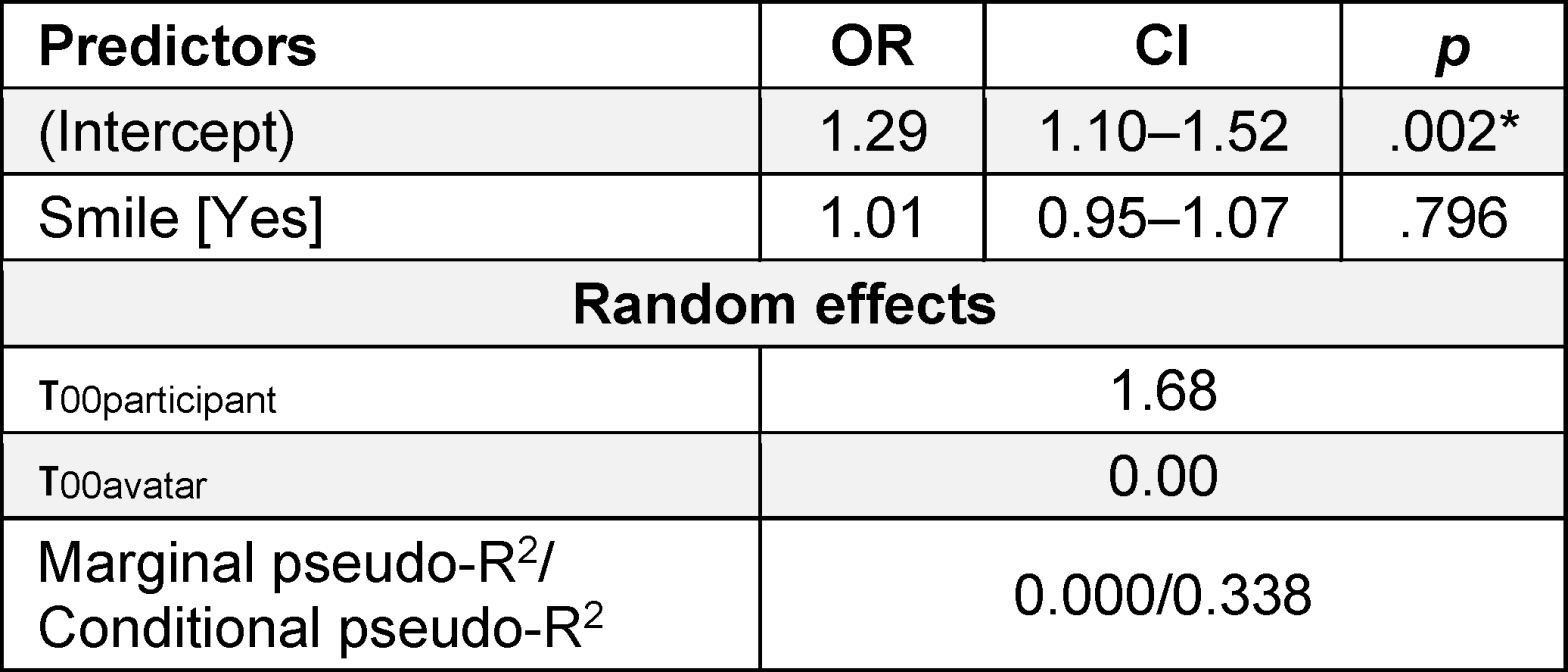
Impact of a Duchenne smile on entrustment decisions

**Table 3:**
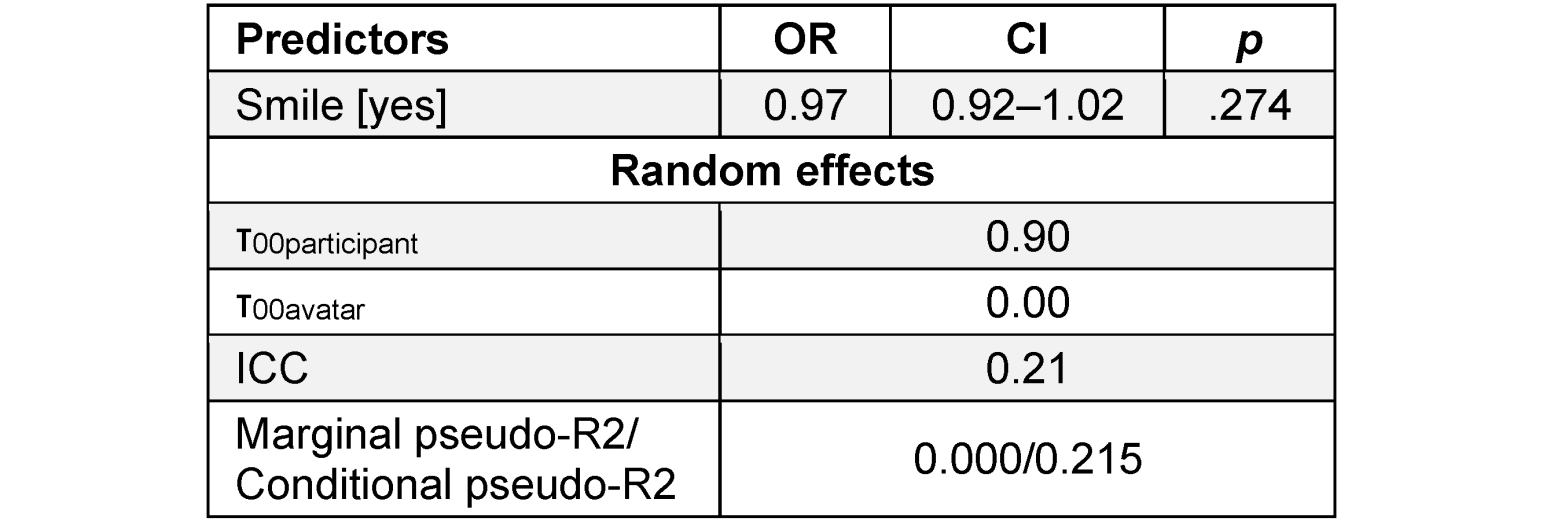
Impact of a Duchenne smile on trustworthiness ratings
